# Locus Reference Genomic sequences: an improved basis for describing human DNA variants

**DOI:** 10.1186/gm145

**Published:** 2010-04-15

**Authors:** Raymond Dalgleish, Paul Flicek, Fiona Cunningham, Alex Astashyn, Raymond E Tully, Glenn Proctor, Yuan Chen, William M McLaren, Pontus Larsson, Brendan W Vaughan, Christophe Béroud, Glen Dobson, Heikki Lehväslaiho, Peter EM Taschner, Johan T den Dunnen, Andrew Devereau, Ewan Birney, Anthony J Brookes, Donna R Maglott

**Affiliations:** 1 Department of Genetics, University of Leicester, University Road, Leicester LE1 7RH, UK; 2 European Bioinformatics Institute, Wellcome Trust Genome Campus, Hinxton, Cambridge, CB10 1SD, UK; 3 National Center for Biotechnology Information, National Library of Medicine, National Institutes of Health, 8600 Rockville Pike, Bethesda, MD 20894, USA; 4 INSERM, U827, Montpellier, F-34000, France; 5 NGRL Manchester, Genetic Medicine, 6th Floor, St Mary's Hospital, Oxford Road, Manchester, M13 9WL, UK; 6 Computational Bioscience Research Center, King Abdullah University of Science and Technology, P.O. Box 55455, Jeddah 21534, Saudi Arabia; 7 Department of Human Genetics, Center of Human and Clinical Genetics, Leiden University Medical Center, Leiden, The Netherlands

## Abstract

As our knowledge of the complexity of gene architecture grows, and we increase our understanding of the subtleties of gene expression, the process of accurately describing disease-causing gene variants has become increasingly problematic. In part, this is due to current reference DNA sequence formats that do not fully meet present needs. Here we present the Locus Reference Genomic (LRG) sequence format, which has been designed for the specific purpose of gene variant reporting. The format builds on the successful National Center for Biotechnology Information (NCBI) RefSeqGene project and provides a single-file record containing a uniquely stable reference DNA sequence along with all relevant transcript and protein sequences essential to the description of gene variants. In principle, LRGs can be created for any organism, not just human. In addition, we recognize the need to respect legacy numbering systems for exons and amino acids and the LRG format takes account of these. We hope that widespread adoption of LRGs - which will be created and maintained by the NCBI and the European Bioinformatics Institute (EBI) - along with consistent use of the Human Genome Variation Society (HGVS)-approved variant nomenclature will reduce errors in the reporting of variants in the literature and improve communication about variants affecting human health. Further information can be found on the LRG web site: http://www.lrg-sequence.org.

## Introduction

In 1993 Ernest Beutler wrote an eloquent letter to the editor of the *American Journal of Human Genetics *highlighting the deficiencies of the systems then used to describe DNA variants [1]. That same year, the editor of *Human Mutation *invited Arthur Beaudet and Lap-Chee Tsui to produce a nomenclature for variants in genes and proteins [2]. From these simple beginnings, the last 17 years have borne witness to the steady development of the nomenclature used to describe sequence variation that is now maintained under the auspices of the Human Genome Variation Society (HGVS) [3,4]. To some, the present nomenclature may seem like an arcane art-form jealously guarded by zealots. This may have been a valid criticism in the past, but advances in human genetics mean that embracing the nomenclature fully is now essential. With the completion of the human genome sequence, the number of known variants has expanded dramatically, with many identified as being associated with medical conditions. For such variants, especially in the clinical setting, the need to describe them systematically is paramount [5-7].

## Reference DNA sequences and their limitations

A crucial element of variant nomenclature is the reference DNA sequence with respect to which a variant is described. Ideally, the sequence should have been submitted to a primary DNA sequence database and be identified by an accession number and its version. For the most part, this requirement is complied with nowadays, though the quality of the sequence data is sometimes questionable. For some genes, the *de facto *reference sequences were established before the advent of high-throughput sequencing technologies. Intron and intergenic sequence data were often less reliable than those of the exons for these legacy sequences due to the deficiency of read-depth coupled with the lack of corroboration of the DNA sequence against a corresponding protein. At the start of the millennium, recognizing the need for higher quality reference data, the National Center for Biotechnology Information (NCBI) established a database of curated non-redundant reference sequences of genomes, transcripts and proteins known as RefSeq [8,9]. Until recently, most human genomic DNA sequences represented in RefSeq have been of genomes rather than individual genes. Consequently, the reporting of variants in genomic DNA coordinates using RefSeq genomic contig sequences has been cumbersome, especially if the gene of interest lies on the reverse strand. For example, the human *COL1A1 *gene is the reverse complement of bases 13535609 to 13553152 in the RefSeq record NT_010783.15. Beginning in 2007, RefSeq has been extended to embrace reference sequences for individual genes through the creation of RefSeqGene records [10]. Many authors still prefer to report intronic sequence variants in terms of cDNA coordinates (for example, c.2451+77C>T, or the now deprecated format, IVS36+77C>T), even though the nomenclature to do so is somewhat awkward. However, the use of cDNA coordinates is permitted with RefSeqGene reference sequences.

In spite of these welcome developments, users of these sequences must be aware of the update policies of public sequence databases. There are two types of modification to a public sequence record: changes to the sequence, or changes to the annotation or description of that sequence. The latter type of change is reflected only by a change to the modification date of the record, and will be changed if the gene symbol changes, citations associated with the record change, or the position of features (such as the coordinates of exons) within that sequence are revised. The former type of change - that is, to the sequence itself - results in the incrementing of the version of the sequence. For example, the sequence of the desmoglein 2 gene, *DSG2*, was revised from version NG_007072.1 to version NG_007072.2 in December 2007. In May 2008, re-interpretation of the mRNA coding regions of the sequence of NG_007072.2 resulted in a change of version number for the corresponding RefSeq mRNA record for *DSG2 *from NM_001943.2 to NM_001943.3 though no change to the RefSeq protein record NP_001934.2 was necessary. The RefSeqGene genomic DNA record and the RefSeq protein record both retained the same version numbers as before, but the RefSeq mRNA record version was incremented. Anecdotal evidence, especially from journal editors, suggests that these issues are poorly understood by researchers who fail to mention the version number of reference sequences that they have used as the basis for reporting sequence variants. Variant reports that do not clearly define the version of the used reference sequence might have ambiguous interpretations.

Failure to fully embrace the issues surrounding versioning of reference sequences can lead to inconsistency of variant descriptions from one generation of patients to the next. An individual testing positive for a given variant today may have children who, years later, wish to seek genetic counseling and be tested for that same variant. To avoid any misunderstanding or confusion by the counselor and the staff of the diagnostic laboratory, it is essential that changes to reference DNA sequences are closely monitored by these parties to militate against the possibility of a change of description for the tested variant (see Box 1 for a hypothetical example).

**Box 1:**
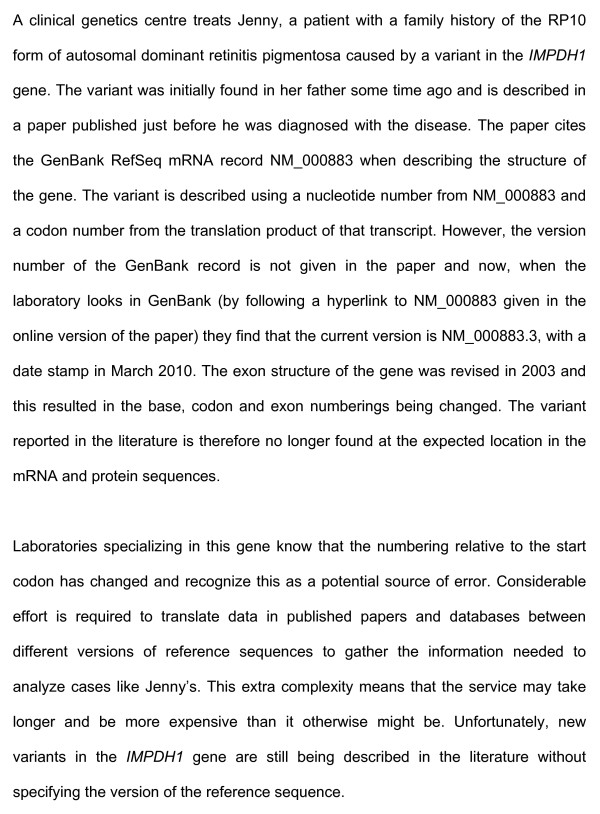
Nomenclature problems because of reference sequence versioning.

Another limitation is that current reference sequences may not represent all transcripts that arise through the use of distinct transcription start sites, alternative splicing, or polyadenylation signals (Box 2). Currently, genomic reference sequences do not necessarily record all of the known mRNAs, focusing instead on information concerning the single most abundant mRNA. Ideally, a reference sequence for a gene would include all relevant spliced transcripts necessary for variant reporting, reducing the risk that an effect of the variant on an alternatively spliced transcript might be missed.

**Box 2:**
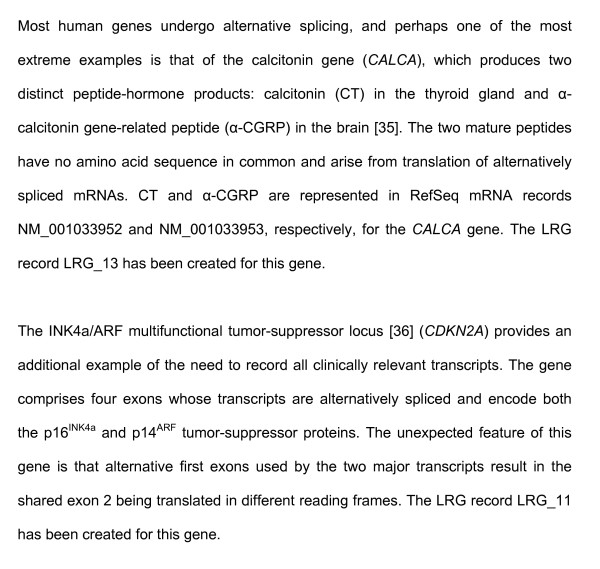
Genes with multiple spliced transcripts.

A further limitation is that the present annotation scheme does not take account of well-established legacy numbering schemata that are in use at present, or have been used in the past, to number features such as exons or amino acids. The globins and the collagens provide excellent examples of legacy systems (Box 3). Ideally, reference sequences would be annotated in a fashion that would allow for verification of variants reported using either legacy or HGVS-compliant nomenclatures.

**Box 3:**
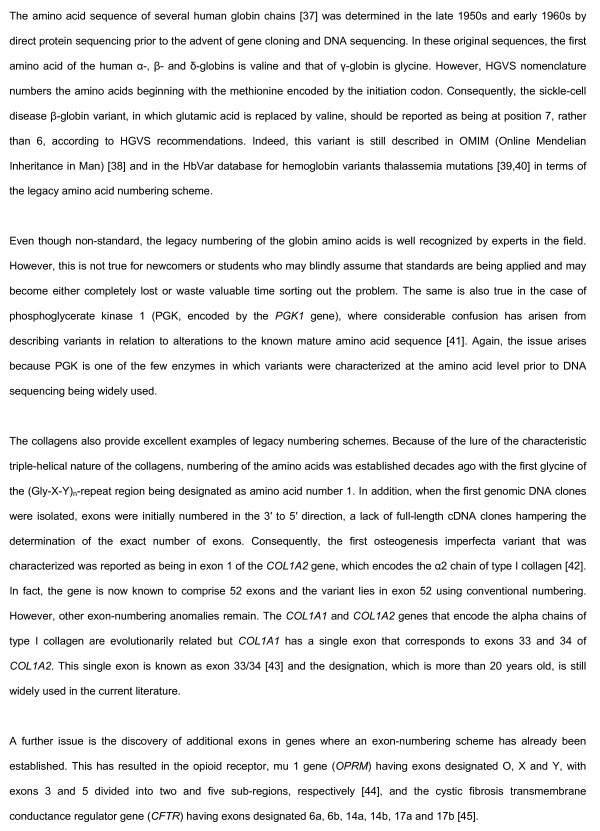
Legacy numbering schemata.

To address these limitations, a meeting sponsored and organized by the multi-institute European Union-funded GEN2PHEN project [11] was held, with representatives of GEN2PHEN (Genotype-To-Phenotype Databases Project), European Bioinformatics Institute (EBI) [12] and NCBI [13] in attendance, to discuss the specification for stable reference genomic DNA sequences better suited to the task of reporting variants. The participants were geneticists, bioinformaticians, clinicians and Locus-specific Database (LSDB) curators. In advance of the meeting, a survey was conducted by GEN2PHEN with the help of the HGVS [14] to assess the views of the curators of LSDBs.

The primary goal of the meeting was to create a universally acceptable standard: a new specification for human genomic DNA reference sequences that would address the shortcomings of non-standardized reporting resulting from a variety of issues. These include the lack of universally agreed genomic reference sequences for some genes even though mRNA, expressed sequence tag and genomic assembly records already exist. Sometimes, there are DNA sequence inconsistencies between existing *ad hoc *genomic reference sequences (where no RefSeqGene record has yet been created) and their NCBI RefSeq mRNA sequence counterparts. Inconsistent and incomplete (and sometimes outdated and inappropriate) annotation of existing *ad hoc *reference sequences and missing annotation of clinically relevant transcripts is an additional problem. Present sequence record systems do not provide support for legacy amino acid and exon numbering schemes. Finally, the new standard needed to address the lack of understanding of the significance of 'versioning' of sequence data.

The principles guiding the discussions with respect to the specifications for genomic reference sequences were threefold. First, the sequences need not represent real alleles of genes: they can be composites that provide a practical working framework for the reporting of variants. Second, research or diagnostic laboratories, LSDB curators, mutation consortia, and so on that have a direct interest in given genes will have the final say in defining the sequences and their annotation. Third, stability of the sequences, their core annotation, and their identifiers is essential to ensure consistency of variant reporting over time frames of many decades.

The agreed solution was the concept of an LRG (Locus Reference Genomic) [15], which builds on the initial ideas from NCBI for RefSeqGene. LRGs will only be created in response to demand from the community which, in practice, is likely to be from LSDB curators or from diagnostic laboratories. LRGs are not restricted to protein coding genes, but will be created for any region of the genome within which sequence variation needs to be recorded, including regulatory regions that encode RNAs. However, the mitochondrial genome is explicitly excluded as its sequence (RefSeq NC_012920.1) and variation is already managed by MitoMap [16]. The LRG system provides a genomic DNA sequence representation of a single gene that is idealized, has a permanent ID (with no versioning), and core content that never changes (that is, nucleotide sequence, transcripts, exons, start and stop codon positions). This core annotation will be known as the 'fixed-annotation layer'. Although LRGs are created for single genes, some might encompass all, or part, of overlapping or adjacent genes, as currently happens with RefSeqGene records. The *LEPRE1 *(leucine proline-enriched proteoglycan (leprecan) 1) LRG (LRG_5) also includes part of the *C1orf50 *(chromosome 1 open reading frame 50) gene, which is encoded on the opposite strand. A separate LRG will be created for *C1orf50 *if there is a request from the community.

Additional annotations, known as the 'updatable-annotation layer', that may change with time (each item carrying its own date stamp) will provide ancillary information about a gene. Such annotations will include details of additional transcripts and information for mapping the LRG sequence onto genome assemblies (for example, currently NCBI 36 and Genome Reference Consortium Human (GRCh) 37) as well as cross-referencing of features in the fixed-annotation layer to legacy coordinate systems.

More than one LRG can be created for a region of interest, should the need arise. If essential changes to any of the core sequence data are required, such as the need to include a newly discovered upstream exon, a new LRG record will be generated with a new ID. Sequence variants may be validly expressed with reference to the original LRG (which will not be retired) or to its replacement. An LRG provides a stable sequence and numbering system against which samples can be compared and variation be reported. Although annotation is provided, the LRG is not intended to aggregate and report all known variants.

Variation will be reported using HGVS nomenclature [4] and the use of an LRG as the reference standard supports all coordinate systems: using genomic DNA coordinates, LRG_1:g.8463G>C is equivalent to NG_007400.1:g.8463G>C; using coding DNA coordinates, LRG_1t1:c.572G>C is equivalent to NM_000088.3:c.572G>C; using protein coordinates, LRG_1p1:p.Gly191Ala is equivalent to NP_000079.2:p.Gly191Ala. As a feature of the LRG project, the coordinate system of a RefSeqGene that matches an LRG will be so indicated and will not be changed.

LRGs afford three key improvements in comparison with RefSeqGene records. LRGs provide a 'one-stop' sequence record for a gene (with a single accession number) comprising sequences for the gene itself, all of the transcripts essential for the reporting of sequence variants, and the corresponding predicted proteins translated from each transcript. The locking of the sequences within the LRG means that 'version-control' is not an issue in the reporting of sequence variants. No sequence (genomic DNA, mRNA or protein) within the fixed layer will ever be changed or removed. Finally, the inclusion of the necessary data facilitates reference to features, such as exons or amino acids, using legacy numbering or naming schemes.

## Implementing LRGs

NCBI continues to identify genes of clinical interest and create RefSeqGene records [10]. In March 2010, RefSeqGene sequences were available for more than 2,800 genes and many of these are already in use in LSDBs. To ease the transition towards the use of LRGs, they will be created from any pre-existing RefSeqGene record. The goal is to maximize the similarities. When a RefSeqGene record is assigned an LRG accession, it means that the sequence, transcripts, proteins and exons are identical for that version of the RefSeqGene and the LRG. In other words, it will make no difference if variants are reported in LRG or RefSeqGene coordinates.

XML was chosen for exchanging and storing LRGs because of its ease of extensibility and validation as well as its natural hierarchical structure, which lends itself well to the nature of the information in LRGs. There are numerous existing programmatic tools for defining, validating, parsing and transforming XML. The XML schema is defined using Relax NG [17] and is available on the EBI FTP site [18]. XSLT style-sheets have been produced that will transform the XML into more user-friendly HTML or plain text. These are also available from the FTP site.

The LRG file has separate XML sections for the fixed-annotation and updatable-annotation layers. Within the tags for the fixed layer are the genomic sequence and transcripts that define the LRG, together with the corresponding cDNA sequence, amino acid sequence and exon coordinate markup. The updatable section contains database cross-references, reports of any overlapping LRGs, detailed information on how the LRG maps to the human genome assembly and information to map systematic exon and amino acid coordinates onto their legacy equivalents.

LRGs will be compiled and maintained by the NCBI and EBI. This will ensure that the data contained in LRGs are accurate and consistent with data in other existing sequence records. The key involvement of these organizations means that the LRG format has an assured long-term future on which users can rely beyond the end of the GEN2PHEN project.

## Downloading and viewing LRGs

The LRG website [15] provides access to existing LRG records and mechanisms for requesting new LRGs. Before making a request, it is advisable that users familiarize themselves with the complete LRG specification [19], which is available on the LRG website, and feedback is invited on any aspect of the specification.

To facilitate viewing of LRGs, Ensembl [20,21] has adapted their browser. NCBI supports displays of LRG sequences and reported variants using client software (NCBI Genome Workbench [22]) and the graphical sequence viewer [23]. Use of these tools facilitates integration of LRG data variant data in dbSNP (NCBI Database of Genetic Variation) [24,25]. The NGRL Universal Browser (National Genetics Reference Laboratory, Manchester;) already provides a graphical view of LRGs [26] with the ability, for some genes, to display tracks linked to dbSNP and to appropriate LSDBs.

A major issue with variant curation is how DNA sequences might be visualized to make the process simpler and less prone to error. Journal editors and referees are well aware of the frequency with which authors report variants erroneously. Ideally, a browser will be developed that will integrate fully with the commonly used LSDB systems, such as LOVD (Leiden Open Variation Database) [27,28], UMD (Universal Mutation Database) [29,30] and MUTbase (Maintenance and Analysis of Mutation Databases on the World Wide Web) [31,32], allowing curators and submitters to automatically generate standards-compliant variant descriptions using the LRG sequences as a reporting reference standard.

Tools such as Mutalyzer [33,34], both in its standalone form and through the API used by the LOVD variant database system, have made the process of correctly naming variants relative to all annotated transcripts and protein isoforms much simpler, but more sophisticated variant-visualization systems would be a welcome development. Just as with Mutalyzer, such systems would parse the annotated features of reference DNA sequences to provide the necessary visual cues to help generate an HGVS-nomenclature-compliant description of any variant. Ideally, such a system would incorporate cross-checking with legacy numbering systems. To achieve this, a robust and comprehensive feature-annotation scheme with a controlled vocabulary will be essential. The LRG specification does not specifically entail the production of a dedicated sequence browser, but producing one that uses LRG sequences would facilitate the successful adoption of LRGs. To this end, the LRG XML schema is fully open and has version control, allowing any party, commercial or public, to develop visualization tools.

## Closing remarks

The LRG specification is the culmination of considerable debate among those participating in the project and has also been fashioned by the advice of external commentators. Most of the proposals have been accepted readily, but two in particular have been controversial. The first is the proposal to allow addition of transcripts to the fixed-annotation layer. The argument is that this amounts to versioning and does not solve the existing version problem. Versioning is an issue with traditional reference sequence records because the actual sequences differ from version to version for records with the same accession number. In the fixed-annotation layer of the LRG, the sequence data for the genomic DNA, the transcripts and their translation products will never be changed or removed. Consequently, a variant description such as LRG_13:g.8290C>A will always remain valid and will not be subject to misinterpretation.

Likewise, the proposal to allow more than one LRG for the same gene region has also provoked similar arguments about versioning. If it is no longer possible to describe a sequence variant in terms of an existing LRG, it might be necessary to create a totally new LRG with a uniquely different number (for example, LRG_1275 instead of the existing LRG_89). The original LRG will not be 'retired' and it will remain valid to describe variants with respect to that sequence record. Creation of additional LRGs for an existing gene or genomic region will only be considered in the most exceptional circumstances and each will be cross-referenced with the other in the updatable annotation layer.

Finally, queries have been raised about the ability of LRGs to support the reporting of copy number variation (CNV). LRGs are no less well suited to the task of CNV description than existing reference sequence records. Requests will be considered for the creation of a new LRG representing a particular allele with respect to CNV and we will work with the requesting party to achieve the best practicable solution to represent the allele. Again, this will only be considered in the most exceptional circumstances. The issues that have been raised during the development of the LRG specification are the subject of a frequently asked questions (FAQs) page, accessible from the LRG home page.

In the absence of any proposals of alternative solutions to deal with these issues, we feel that LRGs provide a pragmatic solution to the needs of LSDBs and clinical laboratories with respect to reporting sequence variants in a stable fashion.

## LRG timeline

Initial discussion of the need for improved reference sequences suited to the task of curation of variants in LSDBs took place at the first general assembly meeting of the GEN2PHEN project in January 2008. Immediately following that meeting, a survey was distributed to LSDB curators through the HGVS and the results were analyzed in March 2008. In April 2008, a two-day workshop was held at EBI to formulate the specification of an improved reference sequence that is now known as Locus Reference Genomic (LRG). Creation of the formal LRG specification began in May 2008 and several versions were produced in response to internal discussion and to feedback elicited through the HGVS. The current version (version 12) was agreed in June 2009.

Creation and revision of the LRG XML schema began in March 2009 (currently at version 1.6) and the first LRG records were created in June 2009. At present, LRGs have been finalized for ten genes and a further four await final approval. Requests have been received for approximately 90 additional genes and these are currently in production. We invite enquiries concerning the creation of additional LRGs.

## Availability

Access to further information and to LRG sequence records is available at [15]. A search facility is provided and there is a link to frequently asked questions (FAQs). Specific links are provided to request technical support, to request the creation of new LRGs and to allow feedback on the LRG specification.

## Abbreviations

CNV: copy number variation; EBI: European Bioinformatics Institute; GEN2PHEN: Genotype-To-Phenotype Databases Project; HGVS: Human Genome Variation Society; LOVD: Leiden Open Variation Database; LRG: Locus Reference Genomic; LSDB: Locus-specific Database; NCBI: National Center for Biotechnology Information.

## Competing interests

The authors declare that they have no competing interests.

## Authors' contributions

The manuscript was drafted by RD with contributions from FC, GP and AD. The manuscript was edited by RD, PF, FC, CB, HL, PEMT, JTdD, AD and DRM. RD, PF, CB, HL, PEMT, JTdD, EB, AJB and DRM participated in the conceptual basis and design specification of LRGs. AD gathered and represented the views of a group of potential users and developed an illustrative example of the requirement for LRGs. GP and FC defined and implemented a format for LRG files. PF, FC and DRM coordinated implementation of the LRG informatics infrastructure. AA, RET, WMM, YC, and PL implemented the software and infrastructure for the project. GD developed the NGRL LRG browser. PEMT and JTdD tested the LRG format for compatibility with existing software tools. BWV designed and implemented the LRG website. RD wrote the FAQs on the LRG website.
